# The 100 Diatom Genomes Project

**DOI:** 10.1371/journal.pbio.3003947

**Published:** 2026-09-01

**Authors:** Thomas Mock, Gust Bilcke, Eliott Flaum, Shunan Fu, Lilian Hoch, Kevin Moog, Nadine Rijsdijk, Elizabeth C. Ruck, Ian W. Bishop, Carole Duchene, Reuben Gilbertson, Amanda Hopes, Christopher Johns, Francesco Manfellotto, Wade R. Roberts, Nigel Belshaw, Udita Chandola, Peter Chaerle, Olga Chepurnova, Daan Deleu, Sofie D’hondt, Federica Di Costanzo, Omaya Dudin, Serena Flori, Trupti Gaikwad, Agnes Groisillier, Andrea Hall, Pengyu Ji, Louis J. Lavier-Aydat, William H. Lewis, Samuel Menicot, Eveline Pinseel, Eléonore Pottier, Krisztina Sarkozi, Arianna Smerilli, Jan Strauss, Sander Thierens, Andrew Toseland, Yousef Touhami, Robert Utting, Michiel Van Bel, Cock van Oosterhout, Yue Wu, Feng Yang, Erika Allhusen, John J. Bolton, Chris Bowler, Thorsten Brinkhoff, Anja Poehlein, Tim Brovarone, Nansheng Chen, Greg Clark, Matthew D. Clark, Dario Copetti, Zongmei Cui, Beini Deng, Jianbo Jian, Uwe John, Anne D. Jungblut, JiHeon Kang, Jon Bent Kristoffersen, JunMo Lee, Shuya Liu, David G. Mann, Linda Medlin, Vincent Moulton, Jelena Radojicic, Shinya Sato, Rosa Trobajo, Klara Wolf, Norico Yamada, Naihao Ye, Libin Zhang, Yunyun Zhuang, Gautam Dey, Valeria Di Dato, Katherine Helliwell, Marianne Jaubert, Peter G. Kroth, Marina Montresor, Giovanna Romano, Tatiana A. Rynearson, Jayson Talag, Klaus U. Valentin, Flora Vincent, Ross F. Waller, Glen Wheeler, Andrew J. Alverson, Kerrie Barry, LoriBeth Boston, Angela Falciatore, Maria Immacolata Ferrante, Jie Guo, Jane Grimwood, Richard Hayes, Andrei Herdean, Jerry Jenkins, Min Kim, Wiebe HCF Kooistra, Alan Kuo, Anna Lipzen, Nicole Poulsen, Jeremy Schmutz, Leïla Tirichine, Klaas Vandepoele, Frédéric Verret, Wim Vyverman, Igor V. Grigoriev

**Affiliations:** 1 School of Environmental Sciences, University of East Anglia, Norwich Research Park, Norwich, United Kingdom; 2 Key Laboratory of Marine Environment and Ecology, Ministry of Education, Ocean University of China, Qingdao, China; 3 Laboratory of Protistology and Aquatic Ecology, Department of Biology, Ghent University, Ghent, Belgium; 4 Department of Plant Biotechnology and Bioinformatics, Ghent University, Ghent, Belgium; 5 Center for Plant Systems Biology, VIB, Ghent, Belgium; 6 Developmental Biology Unit, European Molecular Biology Laboratory, Heidelberg, Germany; 7 Climate Change Cluster, Faculty of Science, University of Technology Sydney, Sydney, New South Wales, Australia; 8 Department of Biological Sciences, University of Arkansas, Fayetteville, Arkansas, United States of America; 9 Graduate School of Oceanography, University of Rhode Island, Narragansett, Rhode Island, United States of America; 10 Laboratoire de Photobiologie et Physiologie des Plastes et des Microalgues, UMR7141, CNRS, Sorbonne Université, Institut de Biologie Physico-Chimique, Paris, France; 11 B CUBE - Center for Molecular Bioengineering, TUD Dresden University of Technology, Dresden, Germany; 12 Integrative Marine Ecology, Stazione Zoologica Anton Dohrn, Napoli, Italy; 13 Department of Biology, Nantes Université, CNRS, US2B, UMR 6286, Nantes, France; 14 Ecosustainable Marine Biotechnology, Stazione Zoologica Anton Dohrn, Napoli, Italy; 15 Department of Biochemistry, Faculty of Sciences, University of Geneva, Geneva, Switzerland; 16 Marine Microbiome Department, Marine Biological Association, Plymouth, United Kingdom; 17 Department of Biology, University of Konstanz, Konstanz, Germany; 18 Department of Biochemistry, University of Cambridge, Cambridge, United Kingdom; 19 Research Data Management Department, Bernhard Nocht Institute for Tropical Medicine, Hamburg, Germany; 20 Division Bioscience, Department Chemical Ecology, Alfred-Wegener-Institute for Polar and Marine Research, Bremerhaven, Germany; 21 Department of Biological Sciences, University of Cape Town, Rondebosch, Cape Town, South Africa; 22 Institut de Biologie de l’Ecole Normale Supérieure (IBENS), Ecole Normale Supérieure, CNRS, INSERM, Université PSL, Paris, France; 23 Institute for Chemistry and Biology of the Marine Environment (ICBM), University of Oldenburg, Oldenburg, Germany; 24 Department of Genomic and Applied Microbiology & Göttingen Genomics Laboratory, University of Göttingen, Göttingen, Germany; 25 Institute of Marine Biology, Biotechnology and Aquaculture, Hellenic Centre for Marine Research, Heraklion, Greece; 26 School of Biotechnological Engineering, Catholic University of Lyon, Lyon, France; 27 Laboratory of Marine Ecology and Environmental Sciences, Institute of Oceanology, Chinese Academy of Sciences, Qingdao, China; 28 Laboratory for Marine Ecology and Environmental Science, Qingdao Marine Science and Technology Center, Qingdao, China; 29 Arizona Genomics Institute, University of Arizona, Tucson, Arizona, United States of America; 30 Department of Sciences, Natural History Museum, London, United Kingdom; 31 College of Marine Science, University of Chinese Academy of Sciences, Beijing, China; 32 Guangdong Provincial Key Laboratory of Marine Biotechnology, Shantou University, Shantou, China; 33 Biodiversity Change, Helmholtz Institute for Functional Marine Biodiversity, Oldenburg, Germany; 34 Department of Oceanography, Kyungpook National University, Daegu, South Korea; 35 Kyungpook Institute of Oceanography, Kyungpook National University, Daegu, South Korea; 36 Science Division, Royal Botanic Garden Edinburgh, Edinburgh, United Kingdom; 37 Algae Save the World, Consulting, Cardigan, United Kingdom; 38 Department of Electrical Engineering and Electronics, University of Liverpool, Liverpool, United Kingdom; 39 School of Computing Sciences, University of East Anglia, Norwich, United Kingdom; 40 Faculty of Marine Science and Technology, Fukui Prefectural University, Obama, Fukui, Japan; 41 Marine and Continental Waters Programme, IRTA-Institute for Food and Agricultural Research and Technology, La Ràpita, Spain; 42 Adaptation and Diversity in the Marine Environment, Roscoff Marine Station, French National Centre for Scientific Research, Roscoff, France; 43 National Key Laboratory of Mariculture Biobreeding and Sustainable Goods, Yellow Sea Fisheries Research Institute, Chinese Academy of Fishery Sciences, Qingdao, China; 44 Cell Biology and Biophysics Unit, European Molecular Biology Laboratory, Heidelberg, Germany; 45 Biosciences, Faculty of Health and Life Sciences, University of Exeter, Exeter, United Kingdom; 46 US Department of Energy Joint Genome Institute, Lawrence Berkeley National Laboratory, Berkeley, California, United States of America; 47 Genome Sequencing Center, HudsonAlpha Institute for Biotechnology, Huntsville, Alabama, United States of America; 48 Institute for Marine and Antarctic Studies (IMAS), Ecology and Biodiversity Centre, University of Tasmania, Hobart, Tasmania, Australia; 49 Center for AI & Computational Biology, VIB, Ghent, Belgium; 50 Department of Plant and Microbial Biology, University of California, Berkeley, Berkeley, California, United States of America

## Abstract

This Community Page presents the 100 Diatom Genomes Project, which aims to sequence the genomes and transcriptomes of 100 diatom species across major lineages, life forms and ecological strategies. This resource will provide a scalable framework for understanding diatom biodiversity, ecology and evolution, and for investigating their use in biotechnology.

## Teaser

One hundred diatom species have been selected for genome and transcriptome sequencing. The 100 Diatom Genomes Project aims to provide a scalable framework for understanding diatom biodiversity, ecology and evolution, and for investigating their use in biotechnology.

## Introduction

Diatoms, the most species-rich algal division, are found in all aquatic and some terrestrial environments, and often dominate primary production. They contribute ~20% of annual global carbon fixation and drive the biological carbon pump sequestering organic carbon to the deep sea [1], yet current genomic sampling is heavily biased toward a limited number of model species [2]. Recognizing their fundamental role in sustaining Earth’s habitability, the 100 Diatom Genomes Project (100DGP) was established as an international, interdisciplinary project, bringing together 108 researchers from 11 countries to leverage diatom genomics across a broad range of scientific disciplines. Expanding the genome representation across diverse diatom clades, ecological niches and life histories will facilitate high-resolution comparative genomics to trace evolutionary innovations.

## Approaches to studying diatoms in the 100DGP

**Fig 1:**
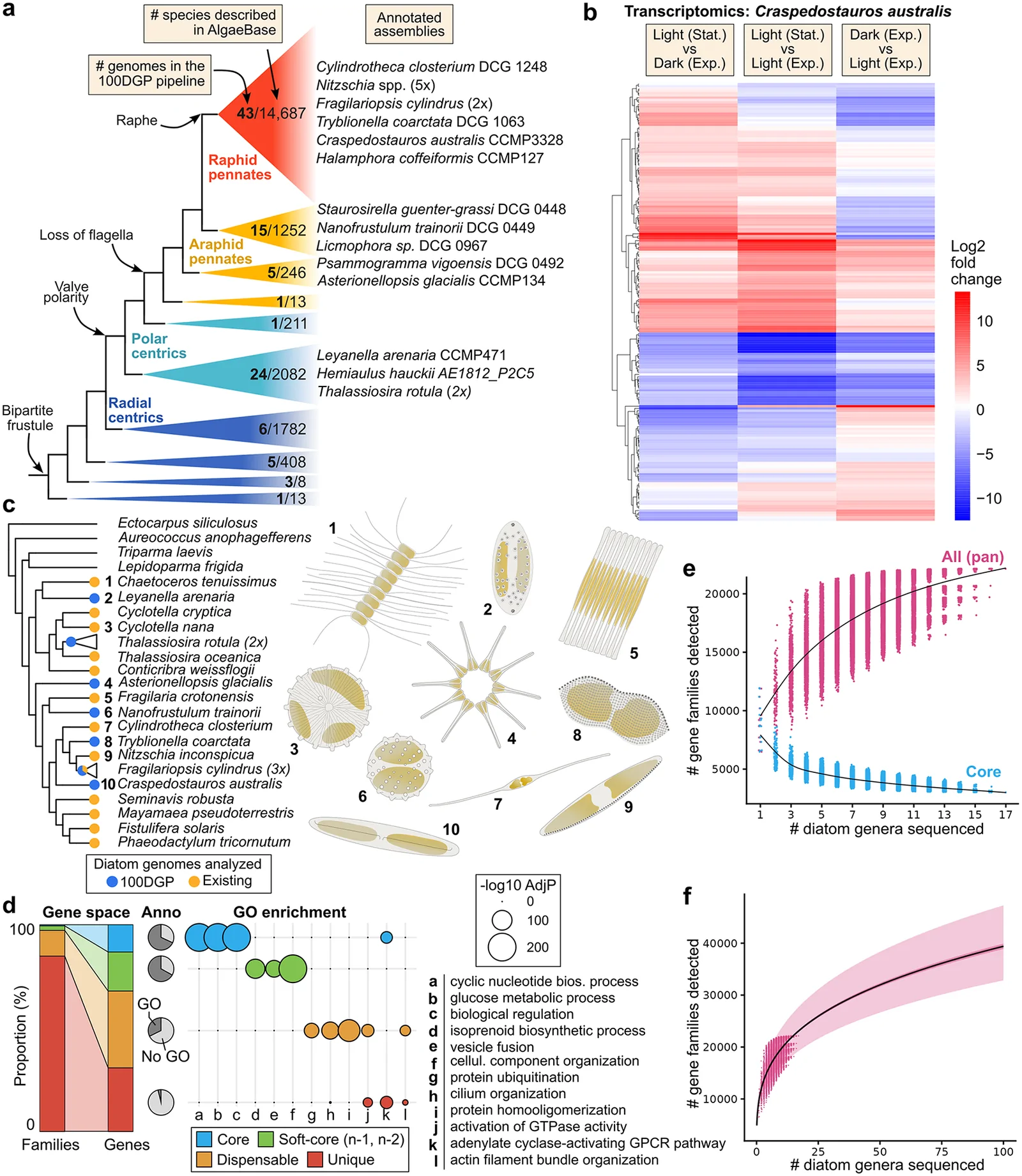
Species selection and gene space diversity of the 100 Diatoms Genome Project. **a**. Simplified drawing of the 10 clades of the diatom phylogeny following [2]. Within each clade, the number of species in the pipeline of the 100 Diatom Genomes Project (100DGP) is indicated, as well as the number of species currently described in AlgaeBase (searched on 3 May 2026). The names of species for which both assembly and annotation of genomes have been completed are shown on the right. **b.** Example of transcriptomic data generated as part of the 100DGP, illustrated for *Craspedostauros australis*. The heatmap shows the log_2_ fold changes of 193 diel-responsive genes identified for cells grown under a 14 h light:10 h dark photoperiod (adjusted P < 0.001; log_2_FC > 2 or log_2_FC < –2). Cells were harvested for RNA isolation at the midpoint of the light phase and the midpoint of the dark phase on day 4, when cultures were growing exponentially (‘Exp.’), and at the midpoint of the light phase on day 12, when cultures had reached stationary-phase and formed a biofilm (‘Stat.’). Gene expression patterns reveal transcriptional responses associated with the diel cycle as well as changes accompanying the transition from exponential growth to stationary-phase biofilm development. **c.** Cladogram of selected diatom and outgroup species used to assess gene space evolution, based on the phylogenetic relationships from [2]. In total, 22 diatom genomes covering 17 genera were selected for a preliminary comparative genomic analysis, combining the 9 first annotated genomes from the 100DGP and 13 publicly available assemblies. Drawings (not to scale) of selected species illustrate the morphological and ultrastructural diversity of diatoms. **d.** Bar plots showing the proportion of gene families with a distinct distribution over diatom genomes: ‘core’, appearing in all diatom genomes; ‘soft-core’, appearing in all but one or two diatom genomes; ‘dispensable’, appearing in 2-19 diatom genomes; and ‘unique’, appearing in a single diatom genome. For each distribution class, pie charts show the proportion that is annotated by at least one gene ontology (GO) term, whereas bubble plots visualize the enrichment significance of the top 3 most enriched GO terms, deduplicated with rrvgo [3]. AdjP: false discovery rate-adjusted p-value. **e.** Pan-genome rarefaction curve [4] showing the number of gene families identified when the number of sequenced diatom genera increases. Both the total number of gene families detected (pink, pan) and the number of families detected in all genera (blue, core) are visualized. **f.** Heap’s law (black line) predicts the expected total number of gene families discovered when an increasing number of diatom genera is sampled. The light pink shade depicts the prediction interval, darker pink highlights the confidence interval.

Species selection was based on practical considerations, including strain availability in culture collections and diatom laboratories from around the world, intrinsic growth rates, and the feasibility of minimizing prokaryotic contaminants. Further checkpoints were the quality and quantity of extractable gDNA and total RNA, both of which can differ substantially between species. So far, representative strains of 104 diatom species have passed our suitability assessments and, of those, 20 annotated assemblies are available (Fig 1a; https://doi.org/10.5281/zenodo.21360381). Although these 104 diatoms cover all major clades, it is not possible to comprehensively sample the entire diversity of diatoms owing to strain availability and cultivability. To mitigate these constraints, we aim to continue the project beyond 100 diatom genomes.

The 100DGP portal provides users with access to all genome assemblies, transcriptomes and annotations. A combination of long-read (PacBio HiFi CCS) and short-read (Illumina NovaSeq X & S4) sequencing has been applied to generate haplotype-resolved genome assemblies and organellar genomes, and a diverse set of transcriptomes (IsoSeq, RNAseq) have been generated for all species for genome annotations. Moreover, for a subset of species, transcriptomes have been generated from cultures exposed to light/dark cycles or raised temperature treatments for future comparative functional studies (Illumina NovaSeq) (Fig 1b; https://doi.org/10.5281/zenodo.21360381). All genomes sequenced at the Joint Genome Institute (JGI) were assembled with hifiasm [5], polished with Racon [6], and annotated using the JGI annotation pipeline [7]. To provide user-friendly interfaces for comparative genomics, multiple bioinformatics analyses were performed and integrated through the PhycoCosm [8] (JGI, Berkeley, USA) and PLAZA platforms (VIB, Ghent, Belgium) (Fig 1c-1f).

**Fig 2:**
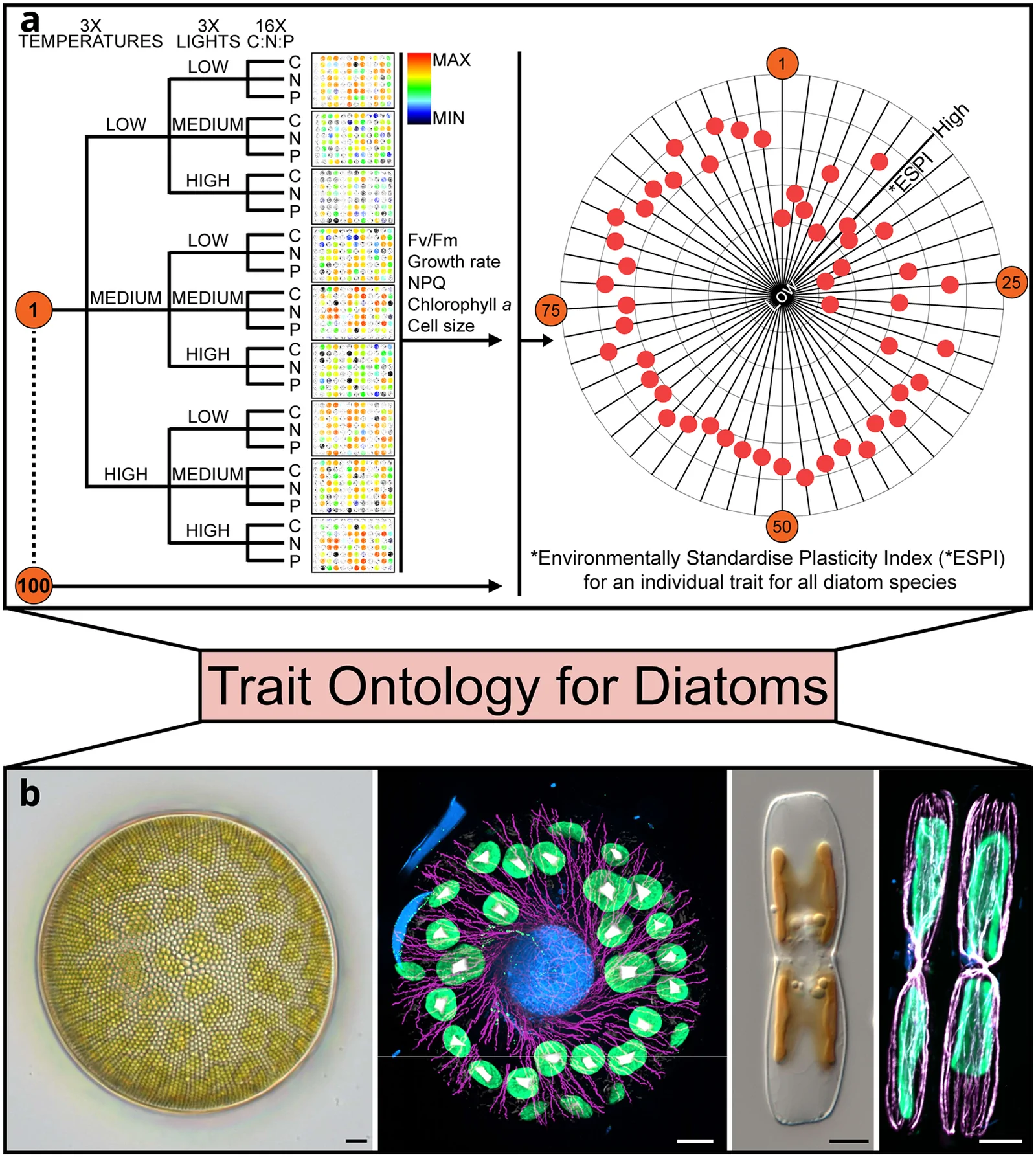
Phenomics, imaging and cellular physiology. **a.** High-throughput phenotyping pipeline to quantify key traits (maximum PSII quantum yield (Fv/Fm), growth rate, cell size, chlorophyl-a content, and non-photochemical quenching (NPQ)), under different environmental conditions (3x temperatures, 3x light intensities, 16x C:N:P ratios). These data will be used to quantify phenotypic plasticity using an Environmentally Standardised Plasticity Index (*ESPI) representing the breadth of the phenotypic responses of each species to changing environmental conditions. This metric is designed to facilitate cross-species comparisons of phenotype plasticity, allowing for greater understanding of phenome response to climate variation. Greatest variance in response per trait is normalized using the Euclidean distance between the environments in which these responses are found, to quantify plasticity per unit of environmental change. In the radial *ESPI plot, each red dot represents the *ESPI value for a given trait for each of the 100 diatom species; larger overall *ESPI values indicate greater phenotypic plasticity. The radial plot shows low *ESPI in the center and high towards the exterior. Information from (**a**) and (**b**) will be merged to develop a trait ontology for diatoms. b. Cell morphology of *Coscinodiscus granii* (left) and *Craspedostauros australis* (right) using light and expansion microscopy (ExM) to demonstrate the nanoscale distribution of molecular complexes: tubulin (magenta), photosystem II (green), nucleus and silica cell wall (blue), NHS ester (white). Black scale bars are 5 μm. White scale bars are 20 μm (4.65 μm biological scale) and 5 μm (1.37 μm biological scale), respectively.

To bridge the gap between genetic variants and key underpinning traits, we are complementing our omics analyses with light and expansion microscopy (ExM) and high-throughput phenotyping (phenomics) (Fig 2). As a technique that enables researchers to physically enlarge diatoms, ExM will facilitate the development of molecular markers to resolve the subcellular localization of proteins with nanometer-scale precision, including those encoded by previously uncharacterized or lineage-specific genes. Our high-throughput phenotyping platform should reveal if and how selected proteins have an impact on measurable traits (e.g., carbon acquisition) and their phenotypic plasticity (Fig 2b). The latter will be quantified using the environmentally standardized plasticity index (*ESPI), a recently developed metric that integrates trait responses across controlled environmental gradients to capture the breadth and magnitude of species-specific plastic responses [9]. By applying the *ESPI, we will compare trait plasticity between species and link differences in their plastic response to ecological niches, global distribution patterns, and therefore adaptive potential. Thus, this integrated framework will lay the foundation for mechanistic trait ontology, which is essential to understand both the evolutionary history of diatoms and the adaptability of important traits.

## Resources and preliminary insights for advancing integrative diatom research

In line with Findable, Accessible, Interoperable and Reusable (FAIR) principles of scientific data management, the 100DGP will publish laboratory methods including standard operating procedures, code, and a diverse set of resources (e.g., genomes, transcriptomes, images, and trait data), together forming an integrated dataset that can be leveraged for future functional studies of diatoms. We encourage researchers to make use of these resources, including the deposited diatom strains at the BCCM/DCG diatoms collection (Ghent, Belgium). Some strains are also available from NCMA (Bigelow, East Boothbay, Maine, USA), RCC (Roscoff, France) and CCAP (Oban, UK). Data integration from genomes, transcriptomes, nanoscale imaging and phenomics is unprecedented for any protist group and will allow us to place comparative evolutionary genomics into the context of cell biology, metabolism and ecology for questions that go beyond the role of individual genes. The first genome assemblies are already publicly available (100DGP portal), revealing a range of genome sizes (75–1,033 Mbp) and >3-fold variation in gene counts. Future comparative analyses will be released through PhycoCosm [8], DiatOmicBase [10], and PLAZA Diatoms: versatile gene-centered platforms for integrative diatom omics research.

External researchers can use this resource to link the outcome of comparative evolutionary genomics (e.g., genomic loci under selection) with nanometer-scale detection of biomolecules and how they shape key traits such as carbon acquisition, nutrient utilization, morphotype diversification, and life-cycle regulation. Beyond fundamental research, our integrative datasets can be exploited for linking genetic variants with the in-vivo function of their encoded proteins and other bioactive compounds for advancing technological applications, such as drug discovery or bioprocess optimization [11].

Preliminary insights based on a comparative analysis of 22 new and existing diatom genomes revealed that only <3% of gene families and 32% of genes are conserved amongst all diatoms (core, soft-core) (Fig 1d). Gene families were identified by performing an all-versus-all homology search between diatom and outgroup proteomes using DIAMOND with subsequent clustering into gene families using the tribeMCL algorithm. Since the rise of diatoms more than 250 mya, a high level of genetic diversity has evolved through a combination of divergent evolution in existing gene families and the appearance of novel genes, either through de-novo gene birth or horizontal gene transfer [12]. Although most gene ontology terms enriched in the diatom shared pan-genome represent genes involved in primary metabolism, the enrichment of cyclic nucleotide-mediated signaling in the diatom core genome supports previous evidence that these secondary messengers drive a multitude of essential abiotic and biotic processes in diatoms. Dispensable and unique genes, on the other hand, were enriched in receptor signaling, flagella organization during reproduction, cell division, DNA recombination, and nuclear organization, which suggest that these processes underpin speciation. Although still preliminary, these results suggest that adaptation may have led to the emergence of novel genes and the exceptional diversity of diatoms.

Based on the current number of genomes, it appears the diatom pan-genome is open (Fig 1e), and Heap’s law [13] predicts that the number of gene families, including their novel genes and variants, will double to ~40,000 by the project end (Fig 1f). To address their potential role in diatom cell biology, particularly if identified as part of known metabolic pathways such as photosynthesis, we will develop molecular markers to reveal the subcellular localization of their encoded proteins using ExM [14]. Our high-throughput phenotyping platform will show if and how those genetic variants and their encoded proteins affect measurable traits, such as light harvesting, carbon acquisition, elemental stoichiometry, or cell division. Early findings indicate that diatoms exhibit a wide range of phenotypic responses, likely driven by their genetic diversity and cellular structural complexity. Altogether, we are constructing a common ontology of traits, critical for developing an integrated approach based on evolutionary genomics, suitable for building predictive models from cell biology to ecosystem science.

## Conclusions

The 100DGP aims to generate diverse biological resources for a key group of primary producers. Our project also aims to provide a test case for protist research at scale, which, for many other protist groups, is still in its infancy. To grow beyond 100 diatom genomes, we are integrating externally sequenced diatom genomes and transcriptomes from other projects (e.g., from the *Chaetoceros* genus sequencing project at JGI). Our preliminary results, based on all resources, provide evidence that integrated research at scale is feasible for diatoms and promise to be a potential treasure trove for discovering novel biology, including unique adaptations and interactions (e.g., endosymbionts). We hope that this work will improve our understanding of global species diversity, evolution, and ecosystem functioning, as well as providing novel genetic variants for advancing biotechnology via genetic engineering and synthetic biology.
